# Urban–rural disparities in education’s mediating role between digital engagement and depression among Chinese older adults

**DOI:** 10.3389/fpubh.2026.1685505

**Published:** 2026-04-10

**Authors:** Guangwen Gong, Haomiao Li, Yuanhang Wang, Yingchun Chen

**Affiliations:** 1 School of Management, Hubei University of Chinese Medicine, Wuhan, Hubei, China; 2 Research Center for the Development of Traditional Chinese Medicine, Key Research Institute of Humanities and Social Sciences of Hubei Province, Wuhan, Hubei, China; 3 School of Political Science and Public Administration, Wuhan University, Wuhan, Hubei, China; 4 Research Center of Health Governance, Wuhan University, Wuhan, Hubei, China; 5 School of Medicine and Health Management, Huazhong University of Sciences & Technology, Wuhan, Hubei, China

**Keywords:** Chinese older adults, depression, digital engagement, education attainment, mediating effect

## Abstract

**Background:**

Chinese older adults face dual challenges of digital exclusion and mental health burdens. This study examines whether educational attainment mediates the relationship between digital engagement and depression, and whether there is urban–rural heterogeneity in this mediating effect.

**Methods:**

Using nationally representative data from 3,206 adults aged ≥60, we conducted chi-square tests and used Hayes’ PROCESS macro (Model 4 for mediation, Model 7 for moderated mediation), adjusting for covariates.

**Results:**

In total, 36.9% (*n* = 1,182 of 3,206) of Chinese older adults reported using the internet. Digital engagement was positively associated with education (*β* = 0.609, *p* < 0.001) and indirectly associated with lower depression through education (indirect *β* = 0.049, *p* < 0.001), accounting for 27.8% of the total effect. A direct association with lower depression remained (*β* = 0.078, *p* < 0.01). The education-mediated pathway showed significant urban–rural heterogeneity, being 29% stronger in urban areas (B = 0.102, 95% CI: 0.077–0.127) than rural areas (B = 0.079, 95% CI: 0.058–0.101; Index of Moderated Mediation = −0.023).

**Conclusion:**

Digital engagement is associated with significant protection against depression among older Chinese adults, and the education-mediated pathway shows substantial urban–rural heterogeneity. To potentially realize the full mental health potential of digitalization, policymakers must implement synergistic strategies to reduce digital infrastructural inequities and address disparities in the translation of education into health benefits.

## Introduction

Depression is a highly prevalent mental illness among older adults. Studies indicate an overall prevalence of 20.0% among individuals aged 60 and above in China (1). By the end of 2024, China’s population aged 60 and above is projected to reach 310.31 million, accounting for 22.0% of the total population. According to the 53rd Statistical Report on the Development of the Internet in China (CNNIC, December 2023), the number of internet users reached 1.092 billion, with 15.6% aged 60 and above (2). This age group represents the fastest growth rate of internet users in the same period (an increase of 2.6%). The rising internet use among older adults, coupled with their high prevalence of depression, has spurred interest in examining the association between digital engagement and depression in this population. The relationship between digital engagement and depression remains unclear. Some studies have reported mixed findings regarding the impact of internet use on depression (3, 4). Certain research suggests digital engagement may lead to social media fatigue, which can significantly increase the risk of depression (5, 6). Conversely, other studies indicate that digital engagement is associated with lower levels of depression in older adults, potentially by enhancing social connectivity and providing online entertainment (7, 8). The differential effects may stem from variations in online activities. For instance, a study based on data from the China Health and Retirement Longitudinal Study conducted in 2018 found that digital engagement was associated with a 37.2% reduction in depressive symptoms among older adults (9). Another study utilizing data from the Chinese General Social Survey (CGSS) in 2018 demonstrated that digital engagement is associated with better self-rated physical and mental health in middle-aged and older adults (10). A US longitudinal study linked health-related digital engagement to slight increases in depression, whereas using the internet for communication with friends and family was associated with slight decreases (11). Existing studies explore associations between different categories of internet use and depression, highlighting online communication’s role in preventing clinical depression among older adults (12). Data from China also show activities such as WeChat chatting, video browsing, and online shopping correlate with lower levels of depression, whereas playing online games and online learning do not appear to reduce depression (13, 14).

Digital technique is changing people’s work and daily lives, traditional social structures, and social forms with unprecedented speed, while older adults do not necessarily keep pace with the rapidly digitalizing society (15–17). This has created a new social governance challenge: the digital divide among older adults (18). Studies indicate that better-educated adults have higher rates of digital engagement and digital skills (19, 20). Limited education can hinder technology access and digital skills, excluding less-educated or uneducated individuals from full participation inthe information society. Consequently, educational attainment is a critical factor in digital engagement and the digital divide (21). The net enrollment ratio of school-age children in China was 84.7% in 1965, indicating that many older adults born before 1965 in China lacked early-life educational opportunities due to the absence of universal compulsory education before the 1980s (22). Older adults without formal education are more likely to be excluded from the information society, which may be linked to their mental health. Therefore, conducting systematic research on how the digital divide, which is closely tied to educational disparities, is associated with the mental health of older adults is of significant importance for promoting healthy aging.

Despite existing studies identifying mediators such as social isolation, social networks, and physical activity in the relationship between digital engagement and depression among older adults (23–25), a fundamental socioeconomic and cognitive resource—educational attainment—has received limited attention as a potential explanatory mechanism. Education established early in life is closely linked to digital access, literacy, and information processing capabilities, all of which may shape the quality and mental health outcome of online activities (26). Thus, a critical gap remains in understanding whether and how educational attainment statistically accounts for the observed association between digital engagement and depression in later life. This gap is particularly salient in the Chinese context, characterized by the world’s most rapid urbanization and widening urban–rural inequalities (27). Significant disparities in both digital infrastructure and educational resources across regions may lead to heterogeneity in the mediating role of education, yet current research lacks a comprehensive analysis of these potential differences. Understanding this nuanced pathway is crucial for developing targeted interventions to bridge the digital divide and its mental health implications. To address these gaps, the present study introduces a resource-enabling perspective. We conceptualize educational attainment not as an outcome altered by digital engagement, but as a pre-existing resource that may enable individuals to derive greater mental health benefits from digital activities. Accordingly, we examine a statistical mediation model (digital engagement → education → depression) to assess whether education transmits part of this association. Therefore, this study aims to: (1) examine the association between digital engagement and depressive symptoms in a nationally representative sample of Chinese older adults; (2) investigate the extent to which educational attainment mediates this relationship; and (3) examine the urban–rural heterogeneity in this mediating effect. Based on our theoretical perspective, we hypothesize that:

*H*1: Digital engagement is associated with lower levels of depressive symptoms.

*H*2: This association is partially mediated by educational attainment.

*H*3: This mediating pathway is stronger in urban areas than in rural areas.

## Methods

### Study design

This cross-sectional study utilized data from the 2021 Chinese General Social Survey (CGSS)conducted by the National Survey Research Center of Renmin University of China. The CGSS is China’s first nationwide, ongoing, large-scale social survey project. The survey employs a stratified four-stage probability sampling method, covering all 2,798 districts and counties nationwide (including 22 provinces, 4 autonomous regions, and 4 municipalities directly under the Central Government; excluding the Xizang Autonomous Region, Hong Kong, Macao, and Taiwan), ensuring strong representativeness. The CGSS is widely recognized as an authoritative data source with high scientific value. For this study, older adults were defined as people aged 60 and above. Respondents under 60 were excluded, yielding an initial sample size of 3,515. Observations with missing or refused responses regarding demographic information were also removed, resulting in a final analytical sample of 3,206.

### Data sources

#### Demographic measurements

Demographic characteristics included sex, age group, marital status, and annual income. In the regression analysis, demographic characteristics were treated as independent variables. Based on the relevant literature (28), these characteristics were included as control variables in the mediation analysis.

#### Depression

Depression, the dependent variable, was measured by the question: “How frequently have you felt depressed or depressed in the past four weeks?” Responses were recorded on a 5-point Likert scale (1 = always, 2 = often, 3 = sometimes, 4 = rarely, 5 = never). For analysis, responses were re-coded: “always” and “often” as 1 (high frequency), “sometimes” as 2 (moderate frequency), and “rarely” and “never” as 3 (low frequency). It should be noted that this single-item measure, while indicative of subjective mood frequency, does not constitute a clinical diagnosis or capture the multifaceted nature of depressive symptomatology as comprehensively as established scales such as the CES-D or PHQ-9.

#### Digital engagement

Digital engagement, the independent variable, was assessed by the question: “What has been your usage of the following media over the past year?” Respondents indicating use of “the internet (including mobile access)” were coded as 1 (users); those reporting use of “newspapers,” “magazines,” “radio and TV,” or “customized mobile news” were coded as 0 (non-users).

#### Education attainment

Educational attainment, the mediating variable, was measured by the question: “What is your current highest level of education?” Responses were coded as follows:

1. Illiteracy (“I have not received any education”).
2. Primary school education (“Private schools, literacy classes,” “primary school”).
3. Junior high school education (“junior high school four”).
4. High school-level education (“Vocational high school,” “Ordinary high school,” “specialized school,” “Technical school eight”).
5. College or postgraduate education (Bachelor’s degree, Master’s degree, or higher).

#### Residential areas

Residential areas, the moderator variable, were measured by the question: “The type of community where the interviewee resides”. Respondents indicating “rural village committees” were coded as 0, and those indicating “urban resident committees” were coded as 1. Refer to Table 1 for detailed variable definitions and codes.

**Table 1:** Variable assignment description.

Variables	Definition/codes
Depression	Always = 1; Sometimes = 2; Rarely = 3
Digital engagement	No = 0; Yes = 1
Education attainment	Illiteracy = 1; Primary school education = 2; junior high school = 3; High school-level education = 4; College or postgraduate education = 5
Residential areas	Urban = 0; Rural = 1
Sex	Male = 1; Female = 2
Age	Age 60 to 69 years = 1; Age 70 to 79 years = 2; Age 80 years and above = 3
Marriage status	Unmarried = 1; Married = 2
Income	Lower than per-capita disposable income = 1; Higher than per-capita disposable income = 2

### Statistical analysis

Data were analyzed using SPSS 24.0 and the PROCESS 3.5 macro. First, chi-square tests were conducted to examine the associations between digital engagement and depression symptoms with key demographic variables (sex, age, marital status, income, educational attainment, and urban/rural residential areas). Second, to test the mediating role of educational attainment in the relationship between digital engagement and depression, we employed PROCESS Model 4, while controlling for sex, age, marital status, income, and residential areas. It should be emphasized that this mediation model examines a resource-transmission pathway for statistical explanation, not a temporal causal sequence. Third, PROCESS Model 7 was applied to examine whether this mediating effect was moderated by urban–rural residence, thereby testing for heterogeneity in the pathway. Finally, a sensitivity analysis was conducted excluding participants aged ≥80 years to assess the robustness of the findings. Several key statistical assumptions underlying the regression-based mediation analysis were examined. The assumptions of linearity and homoscedasticity were checked and deemed tenable through the visual inspection of residual scatterplots. Multicollinearity was assessed by calculating the variance inflation factor (VIF) for all predictors; all VIF values were well below the common threshold of 5 (in fact, below 2), indicating no serious concern. The distribution of residuals was also examined and showed no substantial deviations from normality. Crucially, the significance of the indirect effect was tested using a bias-corrected bootstrapping method with 5,000 resamples. This non-parametric approach does not require the assumption of a normally distributed sampling distribution for the indirect effect, thereby providing robust confidence intervals. For all analyses, statistical significance was set at *p* < 0.05.

## Results

### Descriptive statistics

Sample characteristics are presented in Tables 2, 3. Overall, 36.9% of the participants (*n* = 1,182) reported using the internet, while 63.1% (*n* = 2,204) did not. The sample comprised 51.8% women, 84.9% were aged 60–79 years, 76.4% were married. Based on the 2021 per-capita disposable income (RMB 35,128; ≈US$5,444.9), 63.1% had income below this level, and 51.8% resided in urban areas. Regarding education, 18.8% had no formal education, 30.1% completed primary school, 27.7% completed junior high school, 17.5% completed high school, and only 5.3% attained a college degree or higher. Digital engagement differed significantly across age groups, marital status, income levels, residential areas, and educational attainment (*p* < 0.001). For depressive symptoms, 13.8% (*n* = 442) reported feeling depressed “always” or “often” in the past 4 weeks, 22.1% (*n* = 708) “sometimes,” and 64.1% (*n* = 2056) “rarely” or “never.” Depression prevalence differed significantly by sex, age, marital status, income, and residential areas (*p* < 0.001). Furthermore, both digital engagement and educational attainment varied significantly across depression frequency groups (*p* < 0.001).

**Table 2:** Demographic characteristics and digital engagement among older adults in China (*n* = 3,206).

Characteristics and variables	Digital engagement		*p* value
	No (*n* = 2,204)	Yes (*n* = 1,182)	
Sex			
Male	976	568	0.927
Female	1,048	614	
Age			
Age 60 to 69 years	682	767	<0.001
Age 70 to 79 years	941	332	
Age 80 years and above	401	83	
Marriage status			
Unmarried	556	200	<0.001
Married	1,468	982	
Income			
Lower than the per-capita disposable income	1,616	408	<0.001
Higher than per-capita disposable income	644	538	
Residential areas			
Urban	874	786	<0.001
Rural	1,150	396	
Education attainment			
Illiteracy	534	69	<0.001
Primary school education	756	228	
Junior high school education	505	382	
High school-level education	194	368	
College or postgraduate education	35	135	

**Table 3:** Demographic characteristics and depression among older adults in China (*n* = 3,206).

Characteristics and variables	Depression			*p* value
	Always (*n* = 442)	Sometimes (*n* = 708)	Rarely (*n* = 2056)	
Sex				
Male	161	296	1,087	<0.001
Female	281	412	969	
Age				
Age 60 to 69 years	193	303	953	<0.001
Age 70 to 79 years	184	297	792	
Age 80 years and above	65	108	311	
Marriage status				
Unmarried	305	515	1,630	<0.001
Married	137	193	426	
Income				
Lower than the per-capita disposable income	369	571	1,320	<0.001
Higher than per-capita disposable income	73	137	736	
Residential areas				
Urban	151	331	1,178	<0.001
Rural	291	377	878	
Education attainment				
Illiteracy	125	166	312	<0.001
Primary school education	156	247	581	
Junior high school	109	180	598	
High school-level education	42	97	423	
College or postgraduate education	10	18	142	
Digital engagement				
No	397	494	1,203	<0.001
Yes	115	214	853	

### Mediating effect of educational attainment

**Figure 1:**
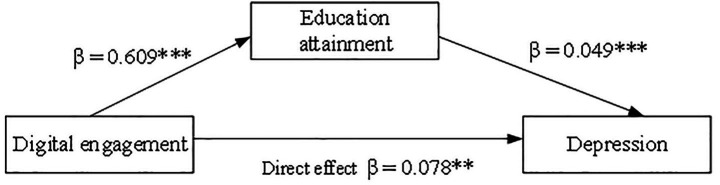
Mediation on model digital engagement to depression on through education in Chinese older adults.

Controlling for sex, age, income, marital status, and residential areas, we use Model 4 to examine the mediating role of educational attainment in the relationship between digital engagement and depression. Bootstrap analysis (Hayes)assessed the indirect effect of educational attainment. The results are summarized in Figure 1 and Table 4.

**Table 4:** Mediation analysis of the effect of digital engagement on depressive symptoms through educational attainment (*n* = 3,206).

Effect type	B (SE)	95% CI	*p*
Total effect	0.108 (0.029)	[0.052, 0.164]	< 0.001
Direct effect	0.078 (0.030)	[0.020, 0.137]	0.008
Indirect effect	0.030 (0.009)	[0.014, 0.047]	—

Figure 1 and Table 4 illustrate the mediating role of educational attainment in the relationship between digital engagement and depression levels, after controlling for sociodemographic characteristics. Digital engagement was positively associated with educational attainment (*β* = 0.609, *p* < 0.001), meaning a one-unit increase in digital engagement corresponded to a 0.609-unit increase in education. Higher educational attainment was significantly associated with fewer depressive symptoms (*β* = 0.049, *p* < 0.001). This indicates that digital engagement indirectly reduces depression by increasing educational attainment. The total effect of digital engagement on depression was 0.108 (*p* < 0.001). Even after controlling for educational attainment, digital engagement remained directly associated with lower depression (*β* = 0.078, *p* < 0.001). The indirect effect *via* education was significant (*β* = 0.030, *p* < 0.001), accounting for 27.8% of the total effect. These findings suggest that educational attainment partially mediates the association between digital engagement and depression among Chinese older adults. This consistent mediation pattern indicates that digital engagement promotes higher educational attainment, which, in turn, contributes to reduced depression.

### Moderated mediation results

We use Model 7 to test for urban–rural heterogeneity in the mediating effect. The results are summarized in Tables 5, 6.

**Table 5:** Urban–rural differences in the impact of digital engagement on education.

Path	B	SE	*p*-value	95% CI
Stage 1: Education				
constant	2.473	0.033	<0.001	[2.407, 2.539]
Digital engagement	0.974	0.049	<0.001	[0.879, 1.070]
Residential areas	−0.428	0.045	<0.001	[−0.516, −0.341]
Digital engagement × Residential areas	−0.218	0.076	0.004	[−0.367, −0.069]
Stage 2: Depression				
constant	2.201	0.032	<0.001	[2.139, 2.263]
Digital engagement	0.087	0.028	0.002	[0.031, 0.144]
Education	0.104	0.012	<0.001	[0.080, 0.128]

**Table 6:** Moderated mediation results.

Variable	Indirect effect	Boot SE	95% CI
Urban	0.102	0.013	[0.077, 0.127]
Rural	0.079	0.011	[0.058, 0.101]
Index of moderated mediation	−0.023	0.008	[−0.040, −0.007]

Table 5 shows that digitally engaged participants had significantly higher levels of education compared to non-digital participants (B = 0.9740, *p* < 0.001), and rural residents had significantly lower levels of education than urban residents (B = −0.4282, *p* < 0.001). The effect of digital participation on education varied between urban and rural areas (B = −0.2179, *p* < 0.004). Higher education was associated with significantly lower levels of depressive symptoms, such that each one-unit increase in education corresponded to a significant decrease in depressive symptoms by 0.104 units (B = −0.104, *p* < 0.01). Even after controlling for education, digital participation remained directly associated with lower depression (B = 0.0873, *p* < 0.01). The direction and significance of the mediating effect in Model 7 remained consistent with those in Model 4, indicating that the moderating variable solely influences the magnitude of the mediating effect. Table 6 shows that the pathway through which digital engagement alleviates depression *via* increased education levels is valid in both urban and rural areas. However, the mediating effect of education is 29% stronger in urban areas (B = 0.102) than in rural areas (B = 0.079). The index of moderated mediation was significantly negative (B = −0.023, 95% CI [−0.040, −0.007]), confirming the urban–rural heterogeneity in the mediating role of education.

### Robustness analysis results

Aging increases the prevalence of physiological changes like chronic illnesses, muscular weakness, and cognitive decline (29), potentially making digital engagement more difficult for adults aged ≥80 compared with younger groups. To assess robustness, we conducted sensitivity analysis excluding participants aged ≥80 years (*n* = 484), leaving a sample of 2,722, and retested the mediation model. As shown in Table 7, the direction and significance of the mediation effect remained consistent with the main findings (Table 4), confirming the robustness of the study’s conclusions.

**Table 7:** Results of robustness analysis (*n* = 2,722).

Predictor	Education attainment		Depression	
	*β*	SE	*β*	SE
Constant	2.516	0.136***	2.188	0.115***
Digital engagement	0.592	0.038***	0.063	0.032*
Education attainment			0.056	0.015***
Sex	−0.397	0.035***	−0.121	0.028***
Age	−0.384	0.035***	0.229	0.019
Marriage status	0.093	0.043*	0.112	0.030***
Income	0.789	0.043***	0.092	0.037*
Residential areas	−0.289	0.037***	−0.160	0.030***
R	0.624		0.257	

*** indicates *p* < 0.05, * indicates *p* < 0.01.

## Discussion

This study employed a large, nationally representative sample of Chinese older adults to investigate the mediating role of educational attainment in the relationship between digital engagement and depression, and to examine the urban–rural heterogeneity in this mediating effect.

### Digital divide and digital engagement patterns

Descriptive analyses revealed that 63.1% (*n* = 3,206) of participants were non-users of the internet. While this rate is lower than reported in the 2018 China Family Panel Studies (14), it remains substantially lower than internet adoption rates among older adults (≥65 years) in the United States (approximately two-thirds) (30). These findings indicate increasing digital engagement, yet still a significant digital divide among Chinese older adults. Our findings align with previous research (31, 32), highlighting that digital participation varies significantly by sex, age, income, education level, and urban–rural status. The gender difference may be partly explained by women’s greater emphasis on maintaining family relationships, facilitated by the internet’s communication channels (33). The age difference is due to age-related declines in cognitive function and technology usability barriers, lower adoption among the oldest-old (34). Socioeconomic disparities are evident, as individuals facing economic or educational disadvantages encounter significant challenges in accessing and using digital resources (35). Finally, the pronounced urban–rural gap in digital engagement underscores a critical aspect of China’s digital divide, reflecting deep-seated regional disparities and structural imbalances (36).

### Association between digital engagement and depression

Consistent with previous literature (31), our study identified associations between key demographic characteristics and depression: males, married individuals, and those with higher income exhibited lower likelihoods of experiencing depressive symptoms. Crucially, even after adjusting for potential confounders, digital engagement demonstrated an independent, significant association with reduced depressive symptoms. This finding is supported by a body of evidence (23, 37–41). The internet offers older adults avenues for accessing health information, obtaining social support, maintaining cognitive engagement, rediscovering social roles, expanding participation in social activities, and enhancing entertainment opportunities (42–44). These functions may be linked to mitigating the impact of physical limitations and functional decline, and are associated with lower levels of depressive symptoms. This is particularly relevant in the Chinese context, where cultural stigma often deters older adults from seeking traditional psychotherapy (45, 46), and access to professional mental health resources remains geographically uneven (47). Digital mental health interventions thus represent a promising approach that may help reduce regional disparities in mental healthcare accessibility.

### The mediating role of educational attainment

Our core finding reveals that educational attainment acts as a significant mediator in the digital engagement–depression relationship. Extant research robustly links higher educational attainment to lower depression risk (48–50), potentially through mechanisms such as enhanced social participation and integration (51). Conversely, lower educational attainment is a known risk factor for depressive symptoms in later life (52, 53). Our mediation analysis indicates that education plays a positive mediating role; specifically, higher educational attainment is associated with a stronger statistical link between digital engagement and lower depression. This enhanced capacity may stem from the greater proficiency of more educated individuals in leveraging the internet effectively: they are better equipped to seek out reliable health information, engage in online fitness or cognitive training programs, participate in enriching entertainment, and maintain meaningful contact with family and friends. These activities provide practical tools for health self-management and sustained opportunities for social connection and engagement, both of which are factors associated with lower depression. Eriksson et al. similarly observed that individuals with higher education mobilize online coping resources more effectively during adversity (54). To address the mental health implications of education-related digital divides, targeted digital literacy training programs are essential for older adults with limited formal education. Community-based participatory learning approaches have shown efficacy in enhancing digital skills and confidence among this group (55).

### Urban–rural disparity in the mediation effect

Our finding reveals that the mediating effect of education is 29% stronger in urban areas (B = 0.102) than in rural areas (B = 0.079). This urban–rural disparity in the mediating pathway may be attributed to structural inequalities in digital infrastructure and educational resources. Such disparities are likely associated with a reduced capacity among rural residents to translate digital engagement into tangible mental health benefits, as their ability to fully leverage online resources may be constrained. This interpretation aligns with the contemporary conceptualization of the digital divide in China, which emphasizes persistent “usage gaps” and “awareness gaps” that extend beyond mere physical access. Even when basic internet connectivity is available, older adults in rural areas often face challenges related to digital literacy—including limited skills in discerning reliable health information online and engaging in meaningful digital social interactions. These limitations may hinder the potential for higher education to amplify the mental health benefits of digital participation, a pattern supported by existing literature (56, 57). For instance, inadequate broadband infrastructure in rural regions has been identified as a critical barrier to effective telehealth and digital learning, disproportionately affecting these communities (58). Furthermore, research suggests that the benefits derived from information technology are not uniformly distributed; individuals with higher educational attainment tend to gain more advantages from digital infrastructure, potentially exacerbating existing inequalities (59). To address urban–rural disparities and derive mental health benefits from digital engagement, it is imperative to bolster the development of information infrastructure in rural areas. This can be achieved through targeted measures such as waiving initial installation fees, exempting basic package fees for the first 2 years, eliminating digital device deposits, and providing subsidized data packages for key populations. Concurrently, the development of platforms such as “Xuetang Online” should be prioritized, with the aim of disseminating customized health science popularization through short videos. This initiative is designed to enhance the health benefits derived from digital participation.

## Conclusion

Our findings suggest that digital engagement is associated with a significantly lower likelihood of depression among older adults in China. However, the observed association is partly explained by an education-mediated pathway, the strength of which is significantly weaker in rural areas, likely reflecting underlying digital and educational disparities. Therefore, interventions aimed at improving mental health outcomes should focus not only on improving internet access but also on actively building the necessary digital competencies, particularly among older adults with lower educational backgrounds. Improving their ability to use the internet effectively for health information and social connection could be a key focus area. Core strategies to maximize the mental health benefits of internet use include narrowing the digital resource gap between urban and rural areas and enhancing mechanisms that translate educational resources into positive outcomes. Targeted efforts to reduce the education-related digital divide represent a promising and practical strategy for fostering healthy aging and improving psychological well-being among older adults in China.

### Limitations

While our analyses used appropriate statistical methods and showed that educational attainment mediated 27.8% of the total effect of digital engagement on depression, several limitations warrant consideration. First, depression was assessed via self-reported frequency of depressive feelings, rather than standardized clinical diagnostic scales, which may affect measurement precision. Second, the cross-sectional nature of the data precludes definitive causal inferences; we can establish associations but not causality. Future longitudinal research is needed to explore these dynamic relationships and strengthen causal interpretations. Third, potentially influential covariates, such as baseline health status, functional limitations, and objective measures of social support, were not included in the models. These factors could significantly confound the observed relationships. Fourth, regarding the mediation model, while we theorize education as an enabling resource (digital engagement → education → depression), we acknowledge that alternative causal orderings (e.g., education → digital engagement → depression) are plausible given the life-course nature of education. Our cross-sectional design cannot definitively disentangle these pathways. Future longitudinal studies should test competing mediation models to establish temporal precedence and strengthen causal inference. Fifth, the measurement of digital engagement was restricted to a binary (yes/no) indicator of any internet use in the past year. This operationalization does not distinguish between different types, frequencies, or qualities of online engagement, which may have distinct relationships with mental health. Future studies would benefit from incorporating multidimensional measures of digital behavior to better disentangle these associations. Experimental or quasi-experimental designs (e.g., digital literacy intervention trials) would provide deeper insights into the complex interplay among specific digital engagement patterns, educational attainment, and mental health outcomes.

## Data Availability

Publicly available datasets were analyzed in this study. This data can be found at: http://cgss.ruc.edu.cn/.
